# Single-cell transcriptomic Atlas of aging macaque ocular outflow tissues

**DOI:** 10.1093/procel/pwad067

**Published:** 2024-02-15

**Authors:** Jian Wu, Chaoye Wang, Shuhui Sun, Tianmin Ren, Lijie Pan, Hongyi Liu, Simeng Hou, Shen Wu, Xuejing Yan, Jingxue Zhang, Xiaofang Zhao, Weihai Liu, Sirui Zhu, Shuwen Wei, Chi Zhang, Xu Jia, Qi Zhang, Ziyu Yu, Yehong Zhuo, Qi Zhao, Chenlong Yang, Ningli Wang

**Affiliations:** Beijing Institute of Ophthalmology, Beijing Tongren Eye Center, Beijing Tongren Hospital, Capital Medical University, Beijing Key Laboratory of Ophthalmology and Visual Sciences, Beijing 100730, China; State Key Laboratory of Ophthalmology, Zhongshan Ophthalmic Center, Sun Yat-sen University, Guangzhou 510060, China; State Key Laboratory of Oncology in South China, Collaborative Innovation Center for Cancer Medicine, Sun Yat-sen University Cancer Center, Guangzhou 510060, China; State Key Laboratory of Membrane Biology, Institute of Zoology, Chinese Academy of Sciences, Beijing 100101, China; Beijing Institute for Stem Cell and Regenerative Medicine, Beijing 100101, China; Institute for Stem Cell and Regeneration, Chinese Academy of Sciences, Beijing 100101, China; Beijing Institute of Ophthalmology, Beijing Tongren Eye Center, Beijing Tongren Hospital, Capital Medical University, Beijing Key Laboratory of Ophthalmology and Visual Sciences, Beijing 100730, China; Beijing Institute of Ophthalmology, Beijing Tongren Eye Center, Beijing Tongren Hospital, Capital Medical University, Beijing Key Laboratory of Ophthalmology and Visual Sciences, Beijing 100730, China; Beijing Institute of Ophthalmology, Beijing Tongren Eye Center, Beijing Tongren Hospital, Capital Medical University, Beijing Key Laboratory of Ophthalmology and Visual Sciences, Beijing 100730, China; Beijing Institute of Ophthalmology, Beijing Tongren Eye Center, Beijing Tongren Hospital, Capital Medical University, Beijing Key Laboratory of Ophthalmology and Visual Sciences, Beijing 100730, China; Beijing Institute of Ophthalmology, Beijing Tongren Eye Center, Beijing Tongren Hospital, Capital Medical University, Beijing Key Laboratory of Ophthalmology and Visual Sciences, Beijing 100730, China; Beijing Institute of Ophthalmology, Beijing Tongren Eye Center, Beijing Tongren Hospital, Capital Medical University, Beijing Key Laboratory of Ophthalmology and Visual Sciences, Beijing 100730, China; Beijing Institute of Ophthalmology, Beijing Tongren Eye Center, Beijing Tongren Hospital, Capital Medical University, Beijing Key Laboratory of Ophthalmology and Visual Sciences, Beijing 100730, China; Department of Neurosurgery, Peking University Third Hospital, Center for Precision Neurosurgery and Oncology of Peking University Health Science Center, Beijing 100191, China; Department of Neurosurgery, Peking University Third Hospital, Center for Precision Neurosurgery and Oncology of Peking University Health Science Center, Beijing 100191, China; Beijing Institute of Ophthalmology, Beijing Tongren Eye Center, Beijing Tongren Hospital, Capital Medical University, Beijing Key Laboratory of Ophthalmology and Visual Sciences, Beijing 100730, China; Beijing Institute of Ophthalmology, Beijing Tongren Eye Center, Beijing Tongren Hospital, Capital Medical University, Beijing Key Laboratory of Ophthalmology and Visual Sciences, Beijing 100730, China; Beijing Institute of Ophthalmology, Beijing Tongren Eye Center, Beijing Tongren Hospital, Capital Medical University, Beijing Key Laboratory of Ophthalmology and Visual Sciences, Beijing 100730, China; State Key Laboratory of Ophthalmology, Zhongshan Ophthalmic Center, Sun Yat-sen University, Guangzhou 510060, China; State Key Laboratory of Ophthalmology, Zhongshan Ophthalmic Center, Sun Yat-sen University, Guangzhou 510060, China; Spencer Center for Vision Research, Byers Eye Institute, School of Medicine, Stanford University, Palo Alto, CA 94304, USA; State Key Laboratory of Ophthalmology, Zhongshan Ophthalmic Center, Sun Yat-sen University, Guangzhou 510060, China; State Key Laboratory of Oncology in South China, Collaborative Innovation Center for Cancer Medicine, Sun Yat-sen University Cancer Center, Guangzhou 510060, China; Institute for Stem Cell and Regeneration, Chinese Academy of Sciences, Beijing 100101, China; Department of Neurosurgery, Peking University Third Hospital, Center for Precision Neurosurgery and Oncology of Peking University Health Science Center, Beijing 100191, China; Beijing Institute of Ophthalmology, Beijing Tongren Eye Center, Beijing Tongren Hospital, Capital Medical University, Beijing Key Laboratory of Ophthalmology and Visual Sciences, Beijing 100730, China

**Keywords:** single-cell transcriptomic atlas, macaque, trabecular meshwork, ocular outflow tissue, aging

## Abstract

The progressive degradation in the trabecular meshwork (TM) is related to age-related ocular diseases like primary open-angle glaucoma. However, the molecular basis and biological significance of the aging process in TM have not been fully elucidated. Here, we established a dynamic single-cell transcriptomic landscape of aged macaque TM, wherein we classified the outflow tissue into 12 cell subtypes and identified mitochondrial dysfunction as a prominent feature of TM aging. Furthermore, we divided TM cells into 13 clusters and performed an in-depth analysis on cluster 0, which had the highest aging score and the most significant changes in cell proportions between the two groups. Ultimately, we found that the APOE gene was an important differentially expressed gene in cluster 0 during the aging process, highlighting the close relationship between cell migration and extracellular matrix regulation, and TM function. Our work further demonstrated that silencing the APOE gene could increase migration and reduce apoptosis by releasing the inhibition on the PI3K-AKT pathway and downregulating the expression of extracellular matrix components, thereby increasing the aqueous outflow rate and maintaining intraocular pressure within the normal range. Our work provides valuable insights for future clinical diagnosis and treatment of glaucoma.

## Introduction

As the aging process accelerates, the burden of eye diseases becomes increasingly significant, impacting both public health and socioeconomic factors (Burton et al., 2021; Pascolini and Mariotti, 2012). Glaucoma, as a prominent age-related eye disease, affects approximately 3.54% of the global population aged 65 years and above, with its prevalence increasing with senescence (Jonas et al., 2017). It is estimated that the number of individuals worldwide with glaucoma will reach 110 million by 2040 (Tham et al., 2014), its clinical features include relative or absolute elevation of intraocular pressure and progressive damage to the optic nerve, ultimately culminating in irreparable visual field defects and blindness (Beykin et al., 2021). Owing to the interplay of genetic and environmental factors, the disease is characterized by multiple causative factors and a complex pathogenesis that remains inadequately understood to date.

Glaucoma is a term that encompasses two primary types of the disease: open-angle and angle-closure glaucoma. Notably, primary open-angle glaucoma (POAG) constitutes approximately 70% of all glaucoma cases (Weinreb et al., 2016). Pathological elevation of intraocular pressure is one of its clinical features. The trabecular meshwork, as the primary site of pathology, plays a crucial role in regulating the aqueous outflow rate and intraocular pressure (Stamer and Clark, 2017). While fibrotic changes in the trabecular meshwork have been observed in glaucoma patients’ tissue specimens, the specific cellular composition and specific component alterations remain unidentified (Adams et al., 2018; Stamer and Acott, 2012). Meanwhile, the incidence of glaucoma significantly increases in the elderly population, and some researchers believe that the number and density of trabecular meshwork cells decrease with age, leading to weakened aqueous outflow tissue (Croft et al., 2017; Gold et al., 2013). Other studies have suggested that during the aging process, the accumulation and changes of extracellular matrix (ECM) are crucial factors leading to fluctuations in intraocular pressure. ECM remodeling, involving alterations in its composition and distribution, results in a reduction in the physiological filtration function of the trabecular meshwork (Johnson, 2006; Liu et al., 2018). These findings suggest that aging of trabecular meshwork cells may be a potential inducer of glaucoma, yet the underlying molecular mechanisms remain elusive.

Single-cell transcriptome sequencing is a high-precision genomic technique that allows for the discrimination of tissue and cell heterogeneity, and identification of cellular features (Macosko et al., 2015). While this novel omics approach can expedite the discovery of cellular composition and molecular distinctions within trabecular meshwork tissue, it presents challenges. With the limited number of cells in the trabecular meshwork tissue and the high cell viability requirements of this technique, obtaining sufficient human samples for experimentation is challenging, resulting in relatively few studies on single-cell sequencing related to aqueous humor outflow tissue and limited knowledge of genetic information at the cellular level (van Zyl et al., 2020). Non-human primates, given their close species origin, sharing of over 90% of DNA sequences, and the presence of highly conserved protein sequences with humans, alongside their remarkably similar anatomical features, stand as the most appropriate experimental animal model (Picaud et al., 2019).

In this study, our plan is to perform single-cell sequencing on the aqueous humor outflow tissue of non-human primates in both young and old age groups to create their transcriptome expression profiles. By grouping the experimental animals according to age, we will further characterize the molecular processes and changes associated with aging in the trabecular meshwork. Additionally, we will attempt to elucidate the potential molecular mechanisms underlying the increased incidence of glaucoma with age. Ultimately, our research will provide crucial clues for exploring new therapeutic interventions for glaucoma.

## Results

### Identification of trabecular meshwork cells and cell subtypes via the single-cell sequence

**Figure 1.**
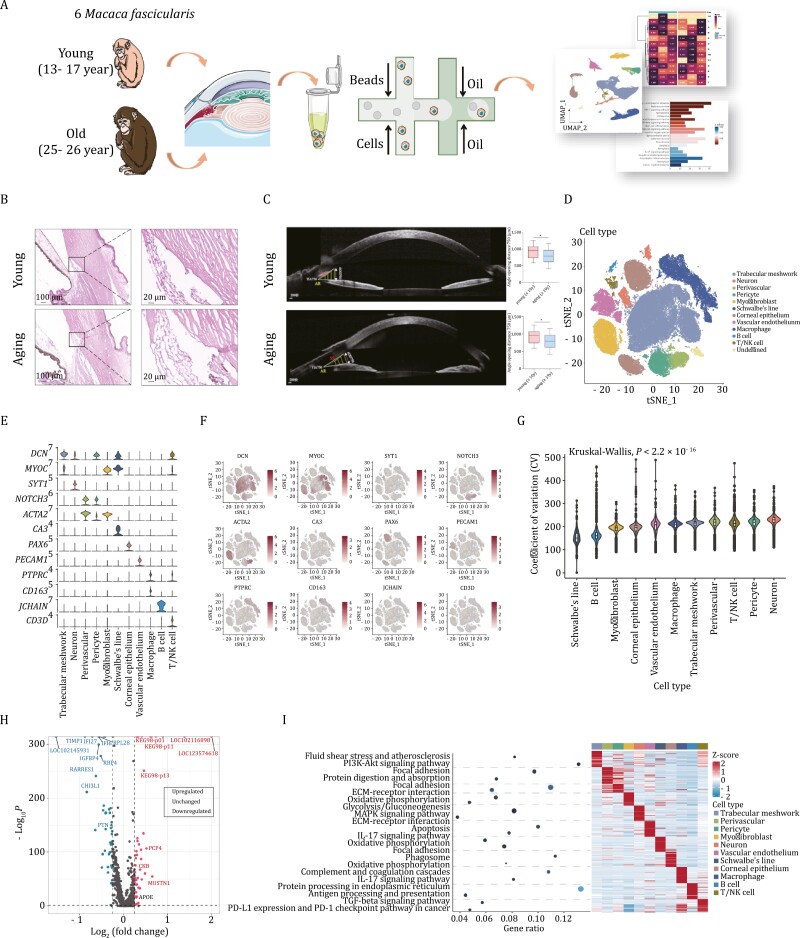
Identification of trabecular meshwork cell types from young and aging samples via single-cell sequence. (A) Experimental workflow for single-cell RNA sequencing of three young and three aging macaque trabecular meshworks. (B) H&E staining of trabecular meshwork of young and aging monkeys. (C) The anterior segment condition of young and aging monkeys was monitored by AS-OCT examination. (D) t-Distributed stochastic neighbor embedding (t-SNE) plot of cell classes from macaque trabecular meshwork labeled by their marker genes. (E) Violin plot showing the expression of different marker genes in diverse cell types. (F) Distribution of the marker genes projected into t-SNE plot. (G) Coefficient of variation showing the correlation between each cell type and aging. (H) Volcano plot for differentially expressed genes (DEGs) of macaque trabecular meshwork, in which *APOE* was among the upregulated ones. (I) Genetic ontology (GO) enrichment analysis results for all cell types from macaque trabecular meshwork.

The function of the trabecular meshwork is regulated by various factors including aging, which can lead to pathological conditions. From a histological perspective, the spindle-like TMCs of the young were arranged regularly while those of the old ones were disordered with decreased and enlarged nucleus (Fig. 1B). Furthermore, according to the anterior segment optical coherence tomograghy (AS-OCT), we observed the aging monkeys exhibited a significant decrease in angle opening distance (750 μm) and trabecular-iris angle 750 (Fig. 1C). However, we still had limited knowledge about the impact of age on the molecular heterogeneity of the trabecular meshwork. Therefore, we decided to perform single-cell RNA sequencing (scRNA-seq) on the trabecular meshwork of 6 *macaca fascicularis*, of which 3 were 13–17 years old (equivalent to 40–50 years old in humans) and the others 25–26 (corresponding to 70–80 in humans). Single-cell suspensions of the monkey trabecular meshwork were prepared by dissecting the eyeball to isolate it from the tissue around the limbus followed by digestion of enzymes, subsequently, we used the 10× genomics platform for sequencing to collect atlases of cells across the samples including seven from the younger ones and seven from the elders (Fig. 1A). DoubletFinder was performed to filter doublet-sand FindIntegrationAnchors was subsequently used to integrate samples and remove batch effects (Fig. S1A and S1B). After quality control, 58,042 cells from aging monkeys and 62,274 cells from younger ones remained.

To uncover heterogeneity within trabecular meshwork, clustering analysis was focused and identified totally 12 distinct classes based on cell type-specific markers described in the published database, shown in the form of t-Distributed Stochastic Neighbor Embedding (t-SNE) (Fig. 1D). Uniform manifold approximation and projection (UMAP) was also conducted for visualization the spatial distance among each cluster better (Fig. S1C). The cell clusters included trabecular meshwork (*DCN*; *MYOC*), perivascular (*NOTCH3*; *ACTA2*), pericyte (*DCN*; *NOTCH3*; *ACTA2*), myofibroblast (*MYOC*; *ACTA2*), neuron (*DCN*; *SYT1*), vascular endothelium (*PECAM1*), Schwalbe’s line (*DCN*; *MYOC*; *CA3*), corneal epithelium (*PAX6*), macrophage (*PTPRC*; *CD163*), B cells (*JCHAIN*), T/NK cells (*DCN*; *MYOC*; *CD3D*) and one undefined cluster with no identity assigned (Fig. 1E). We also organized the cells into UMAP and t-SNE plots based on their cell type annotations (Figs. 1F and S1D). Remarkably, *DCN* and *MYOC* were distributed mostly in TMCs based on the cell types noted in Fig. 1B, suggesting that these two genes were specifically expressed in TMCs thus making it possible to separate it from other cell types. For other marker genes such as ACTA2, it was mainly expressed in the group of myofibroblasts and perivascular cells, the same was applicable for other markers in labeling different cells. Furthermore, we identified the fibroblasts and macrophages using their specific cell marker (NEB and CD206 respectively) by immunofluorescence (Fig. S1E and S1F).

To capture the correlation between each cell type with the aging process, we calculated the coefficient of variation (CV) to identify aging-associated changes in gene expression of cell types and found that neurons took the first place followed by pericyte, T/NK cells, perivascular cells, trabecular meshwork, macrophage and so on (Fig. 1G), indicating the involvement of neuron, immune, inflammation plus TMC degeneration itself along with senescence. Of note, trabecular meshwork cells, perivascular, pericyte, Schwalbe’s line, and vascular endothelium were predominantly enriched in the young group, and the rest of the cells were mainly concentrated in the senescent group (Fig. S1G).

We focused on transcriptional patterns of trabecular meshwork tissues from the young to aging individuals, conducting a differential expression gene (DEG) analysis by using the function of Seurat package. This analysis revealed 90 DEGs, including the upregulation of APOE, as depicted in the volcano plots. (Fig. 1H). And in the biological function of each cluster, we found the gene ontology (GO) terms related to the “PI3K-AKT signaling pathway” were enriched in trabecular meshwork (Fig. 1I). Following this, we isolated the trabecular meshwork cells for GO analysis, we discovered that these genes may play roles in influencing inner mitochondrial membrane protein complex, proton-transporting adenosine triphosphate (ATP) synthase complex, oxidative phosphorylation and so on (Fig. S1H), indicating that the mitochondrial metabolic processes were affected and altered with aging. These results were corroborated through *in vitro* experiments involving primary TM cells. These experiments demonstrated that the ability to produce ATP was impaired with aging but improved when *APOE* silencing (Fig. S5B). In summary, these findings elucidate mainly cell types within the trabecular meshwork and their potential roles in the aging process.

### scRNA-seq revealed TM clusters which played important roles in aging

**Figure 2.**
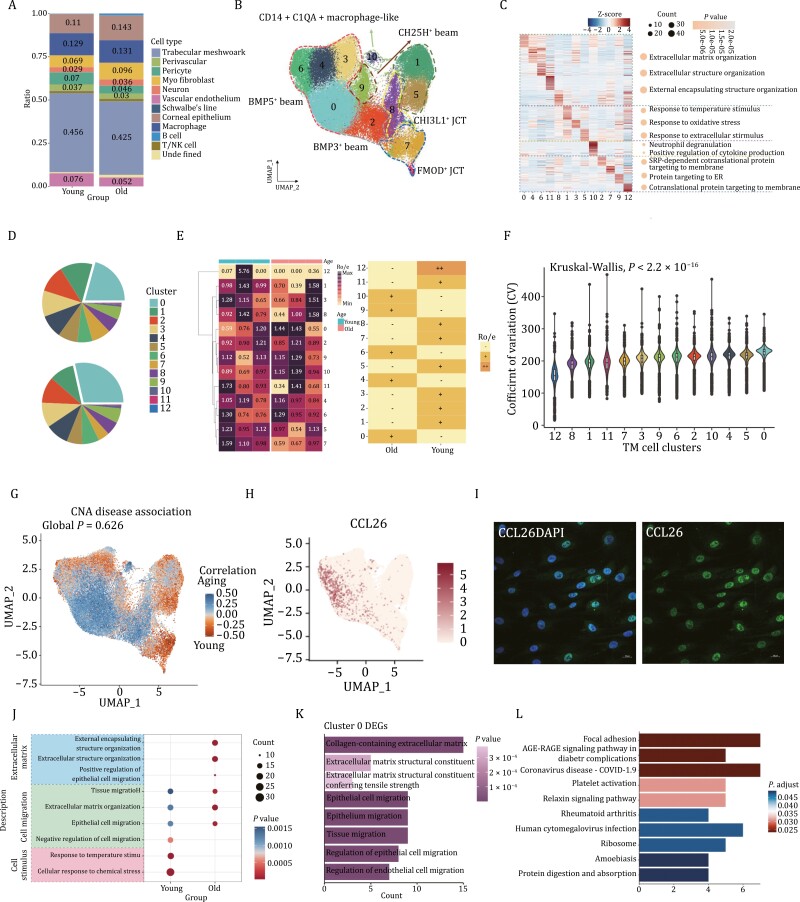
The heterogeneity of trabecular meshwork cell clusters in aging and young eyes. (A) Bar chart showing proportion changes of each cell type with aging, revealing that trabecular meshwork cells decreased significantly in older trabecular meshwork. (B) UMAP plot exhibiting clusters of trabecular meshwork cells distinguished by different markers (dots, individual cells; color, clusters). (C) GO analysis showing different pathways for each cluster performed in aging. (D) Distribution of clusters 0–12 in macaque samples of different ages is shown in pie chart. (E) Heatmap showing the difference of each cell cluster in trabecular meshwork cells, indicating some were enriched in the old group like cluster 0 while some others gathered more in the young group. (F) Coefficient of variation (CV) of TM Seurat clusters suggesting the significant role of cluster 0 in aging. (G) Co-varying neighborhood analysis showing cluster 0, 4, 10, 2, and 6 may exhibit aging tendency. (H) Distribution of the CCL26 in t-SNE plot. (I) Immunofluorescence staining of the cluster 0’s maker CCL26. (J) GO enrichment analysis for trabecular meshwork cells between the young and the old. Color of the dots indicates the significance of corresponding item and size of each dot represents the cell counts. (K) GO analysis of DEGs for cluster 0 showing the effect of collagen-containing extracellular matrix and cell migration in aging. (L) KEGG analysis showing the function of cluster 0.

By comparing the cell composition between the young and the elderly groups, we observed distinct distributional preferences among these cell clusters, as illustrated in Fig. S1E. Notably, the proportion of TMCs, perivascular, pericyte, and vascular endothelium decreased while macrophages, myofibroblast, and neuron increased (Fig. 2A), indicating that aging trabecular meshwork shared heterogeneous cell atlases with youngers. Consistently, the results may give an illustration of the pathological mechanisms of some types of glaucoma such as primary open-angle glaucoma (POAG), whose incidence increased with age. Cause of the significant decreation of cell ratio in aged group as well as the functional and morphology changes in anterior-segement of *Macaca fascularis*, we initially focused on TMCs.

Thus, we next probed to TMCs only and identified 7 beams and 13 clusters using a series of marker genes, including the *BMP5*^*+*^ beam (clusters 0, 3, 4, and 6), *BMP3*^*+*^ beam (cluster 2), *PTN*^*+*^ beam (cluster 1 and 5), *CH25H*^*+*^ beam (cluster 9), *CHI3L1*^*+*^ JCT (cluster 8), *FMOD*^*+*^ JCT (cluster 7 and 10) and *CD14*^*+*^*C1QA*^*+*^ macrophage-like cluster (cluster 10) (Fig. 2B). They were subsequently grouped into the UMAP plot. We next conducted the age-related GO term enrichment analysis in each cluster (Fig. 2C). These cell clusters could be identified into four types based on GO annotation to significant cell marker genes. Clusters 0, 4, 6, and 11 mainly enriched with the pathway of the extracellular matrix which implied the important function of organizing extracellular components. Clusters 1, 3, and 5 were associated with the response of stimulus. Cluster 10 expressed multi-cytokines genes which may take on immune functions and cluster 2, 7, 9, and 12 were correlated with protein synthesis (Fig. 2C). In addition to uncovering the specific cluster and enriched pathways by identifying differentially expressed genes (DEGs), we analyzed the cell ratio of each cluster and captured precise changes of subcluster numbers, which might also be important in aging-related TM diseases and be of significance to determine their targets in the future. To clarify the significance of each cluster of TMCs in aging, we compared the difference of them between young and aging samples and found that cluster 0, as well as cluster 10, may play an important role in the process of senescence, while clusters 7 and 8 seemed to be negatively associated with aging (Fig. 2D and 2E). The obviously increasing cell ratio of cluster 0 was observed. Additionally, CV analysis of major cell clusters was applied to identify the age-associated clusters more directly. Major changes happened in cluster 0 (associated with extracellular matrix) which took the first few spots in aging groups. Interestingly, cluster 10 (macrophage-like cells) was also correlated with the aging group (Fig. 2F). Additionally, Co-varying neighborhood analysis showed that apart from cluster 0, clusters 4, 10, 2, and 6 also exhibited aging tendency while the others may be less or contradictorily related to the aging process (Fig. 2G). Results also screened out three genes including *APOE*, *CLU,* and *IGFBP2*, of which *APOE* expression was approximately concentrated on aging cell distribution (Figs. 2J and S4A–C).

Given that we have focused on cluster 0 in TMC, projected the CCL26 in TMC u-map, and identified the maker of cluster 0 according to the IF (Fig. 2H and 2I). After that, we performed GO analysis for this single cell type between the young and the old, and the results exhibited participation of external encapsulating structure organization, extracellular structure organization, and tissue migration (Fig. 2J). Given the given the upregulation of cluster 0 in aging groups and its possible significance in aging, GO analysis has been done for cluster 0 between youngsters and elders, showing the most upregulated pathways like collagen-containing extracellular matrix, extracellular matrix structural constituent, epithelial cell migration, and tissue migration, consistent with the result for integrated TMCs (Fig. 2K). For the possible roles of other clusters performed in aging, GO and KEGG results were all clarified and shown in Figs. S2 and S3. At the same time, we searched for the functions cluster 0 played, and some pathways related to focal adhesion, AGE-RAGE signaling pathway, coronavirus disease, and so on were revealed (Fig. 2L). These results revealed the alteration of the extracellular matrix and regional immune microenvironment in older *Macaca fascularis*.

### Different gene expression signatures and phenotypic transformation of macrophages with aging

**Figure 3.**
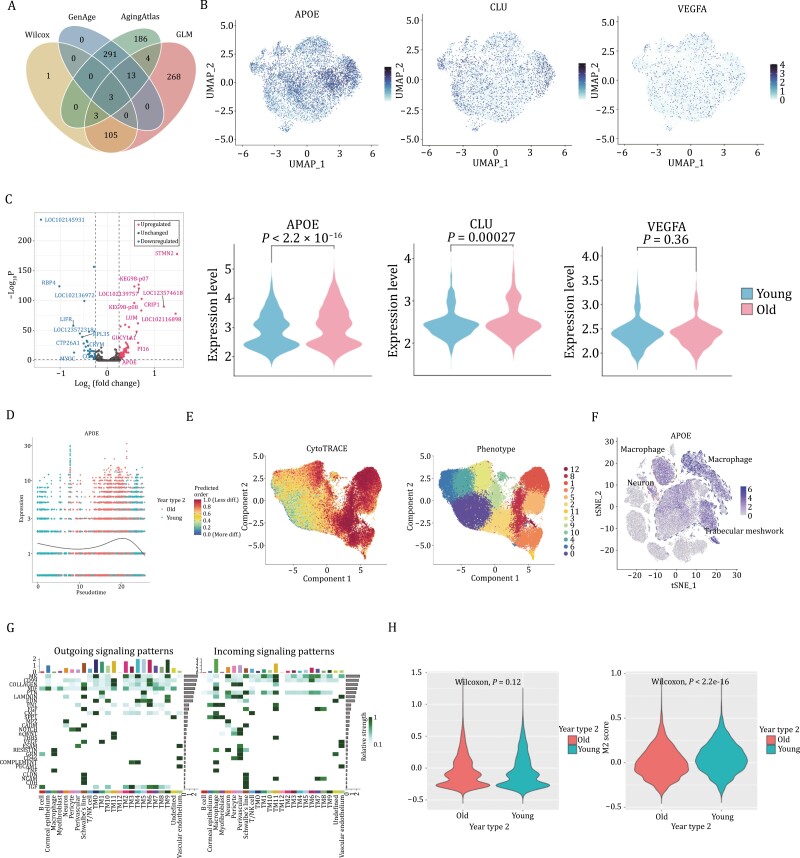
Different molecular expression signatures of cluster 0 as well as macrophages with aging. (A) Venn diagram showing the overlapping gene between upregulated and downregulated DEGs within each dataset. UMAP for visualization of the distribution of three DEGs and corresponding expression levels in young and old respectively. (C) Volcano plot showing upregulated and downregulated genes of cluster 0 in young and aging trabecular meshwork. (D) Pseudotime of cluster 0 with the effect of *APOE* generated by Monocle 3 package of R software. Each dot represents a single cell. (E) The differentiation ability of each cluster analyzed by CytoTRACE, illustrating the cell living activities during different stages. (F) t-SNE plot showing the expression of *APOE* was significantly upregulated in aging trabecular meshworks and macrophages as well. (G) Cluster 0 was in connection with macrophages via CXCL signaling patterns. (H) Violin plot showing different M1 and M2 cell scores between young and aging samples.

To screen the key genes associated with aging in cluster 0, we conducted a comparative analysis of aging-associated DEGs in cluster 0 calculated by the Wilcox rank sum test and generalized linear model with aging-associated genes annotated in the GenAge and Aging Atlas database., and then Venn diagram analysis (VDA) was applied to describe the overlap or nonoverlap of the key performance genes. Finally, 874 assessments were included and cluster 0 in aging cases showed a significant upregulation of *APOE*, *CLU,* and *VEGFA* (Fig. 3A). To effectively test the expression level of these genes on aging Macaque trabecular meshwork sections, we re-integrated cluster 0 cells and projected them onto a UMAP space. By performing quantitative calculations, we were able to assess the relatedness of the three DEGs across the aging process (Fig. 3B). Notably, the expression level of *APOE* was the most prominent, moreover, the significant difference between the young and the old could be seen from the violin plot. The upregulated and downregulated genes labeled in the volcano plot also, reassured the higher expression level of *APOE* in aging individuals (Fig. 3C). We established aging scores using an aging-relevant gene set from Monocle 3 and found that the expression levels of *APOE* and *CLU* showed an elevation trend during different life stage (Figs. 3D and S4D). However, TMCs showed the highest expression level of *CLU* at the age of 10 and it conversely fell off over 10-year-old, suggesting that *CLU* may mainly accelerate the TM aging process in middle age. Also, the expression level of *VEGFA* seemed not significantly correlated with senescence, indicating the different roles these genes played in aging by single-cell transcriptomic profiles (Fig. S4E). Nevertheless, the specific regulons of *APOE* were not within the highly activated regulons when investigating cluster 0 (Fig. S4F), which needs to be further thought and explored. To investigate the aging trajectories of the trabecular meshwork in cynomolgus monkeys, CytoTRACE was used at first to estimate the nature of differentiation with aging, and it came out that cluster 0 was at the end of the differentiation stage (Fig. 3E). Since the cluster of macrophage-like cells was among top clusters in aging samples shown in Fig. 2D also the expression of *APOE* was significantly upregulated in aging macrophages (Fig. 3F), we then explored the interaction of cluster 0 cells and macrophages.

Interestingly, we observed the expression level of *APOE* might not only have an effect on TMCs but also on macrophages via the CXCL signaling pathway, which functioned in inducing inflammatory chemokines and migration of immune cells during infection or injury (Fig. 3G). To be specific, the expression level of CXCL12 saw an increase in older samples compared to the younger one (Fig. S4G). The constitution score of macrophages’ subtypes-M1 and M2- further confirmed that the participation of immune and inflammation with aging since the violin plot saw an upregulation of M1 which mainly initiated an inflammatory response while an obvious downregulation of the anti-inflammatory M2 group (Fig. 3H). In view of the analysis mentioned above, we next built an aging model of macrophage cell line and knocked down the expression of *APOE*, and we found a surprisingly consistent result that phenotypic transformation of macrophage happened when aging but reversed partially by *APOE* silence despite some statistic differences not significant (Fig. S4H and S4I).

### Aging increases the expression of *APOE*, *CLU*, and *VEGFA* in TMCs

**Figure 4.**
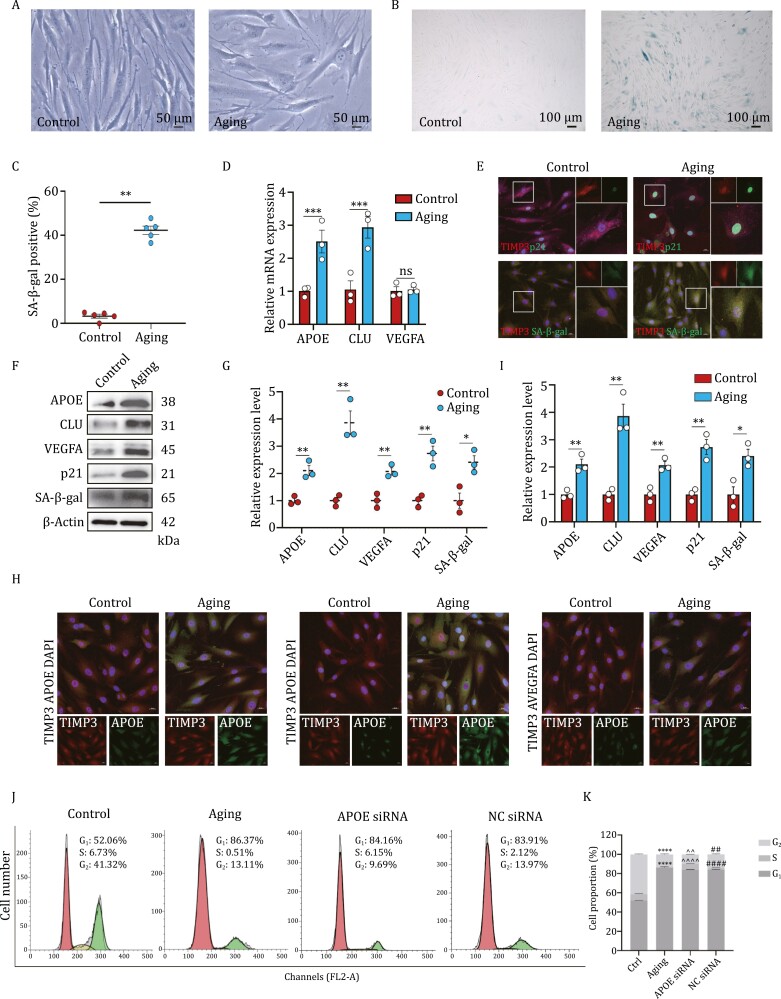
Aging increases the expression level of APOE, CLU, and VEGFA in TMCs. (A) Morphologic changes of trabecular meshwork cells (TMCs) after senescence induction. (B and C) SA-β-gal staining exhibits the increase of aging cells with H_2_O_2_. (D) QRT–PCR was used to detect the mRNA level of three DEGs. (E) Immunostaining of p21 and SA-β-gal in aging TMCs. Scale bars: 20 μm (left). (F) Western blot was performed to detect the protein levels of three DEGs, senescence markers, and β-actin. (G) Quantification of the relative expression level of proteins in (F). (H) Immunostaining of *APOE*, *CLU,* and *VEGFA* in normal and aging TMCs. Scale bars: 20 μm. (I) Quantification of relative fluorescence intensity in (E and H). (J) Flow cytometry was used to detect cell cycle of TMCs with different treatments. (K) Proportion of each cell cycle phase in TMCs at different stage. Data are presented as mean ± SEM values; *P < 0.05, **P < 0.01, ***P < 0.001, ****P < 0.0001; *n* = 3 (*n* = 5 for immunofluorescence).

To prove that the cell strain we studied was from human TM tissue, we used dexamethasone at a concentration of 500 nm to induce the cell strain according to the consensus characteristics of TMCs. The results showed that an increased expression of MYOC after induction, validating the identity of our cultured TM (Fig. S4J and S4K). Given that the morphology and physiological function of aging cells differentiate significantly from normal cells, we compared the appearance of normal and aging TMCs. After H_2_O_2_ induction, the cell number decreased accompanied by TMCs becoming flatter and more enlarged, and the nucleus were enlarged as well (Fig. 4A), typical of senescent cells (Angello et al., 2005; Cristofalo and Sharf, 1973; Kumazaki et al., 1991; Sherwood et al., 1988). Next, we confirmed this method resulted in aging and assayed for senescence-associated β-galactosidase (SA-β-gal), a general marker of senescence (de Jesus and Blasco, 2012; Dimri et al., 1995). The results showed that the percentage of blue-stained SA-β-gal-positive TMCs increased significantly with the existence of H_2_O_2_ (Fig. 4B and 4C), indicating the aging model was established successfully. To determine whether the protein level of senescence markers including SA-β-gal and p21 were also upregulated correspondingly, we surveyed them using immunofluorescence assay. Images showed that aging induction enhanced SA-β-gal and p21 expression post-H_2_O_2_ exposure. (Fig. 4E and 4I). Consistently, Western blot results showed the two protein levels increased in response to H_2_O_2_ stimulation than in their normal state (Fig. 4F and 4G). To monitor the variation tendency of three DEGs distinguished by bioinformatic analysis between normal cells and senescence-inducing cells, the mRNA levels of *APOE*, *CLU*, and *VEGFA* were investigated at first and the qRT-PCR results demonstrated that *APOE* and *CLU* but not *VEGFA* were elevated significantly in the aging group (Fig. 4D). We then turned to investigate the protein levels of three DEGs. Their increases were proved through a Western blot (Fig. 4F and 4G). We stained the primary TMCs of control and treated groups with antibodies for *APOE, CLU,* and *VEGFA* as well as TM tissue marker gene *TIMP3*, and surveyed them using immunofluorescence, confirming that aging TMCs expressed more *APOE, CLU*, and *VEGFA*, consistent with the findings of Western blot (Fig. 4E and 4I). Collectively, these results suggested that *APOE*, *CLU*, and *VEGFA* played a role in TMC aging.

### Knockdown of *APOE* rescues aging-induced cell apoptosis and molecular function damage partly via the PI3K-AKT signaling pathway

Considering the significant upregulation of *APOE* confirmed by different experimental methods, we then proceeded to investigate the potential mechanisms of *APOE* to accelerate trabecular meshwork degeneration in aging. We knocked down the expression of *APOE* in TMCs by transfecting *APOE* siRNA into TMCs for 24 h, followed by stimulation of low-dose H_2_O_2_ to induce the phenotype of aging. To offset the toxicity of siRNA and transfection agent, another group of TMCs was transfected with negative control (NC) siRNA. The transfection efficacy is shown in Fig. S5C. After different disposal, cell cycle analysis using flow cytometry was conducted to detect the proportion of cells in the synthesis (S) phase, gap phases (G_0_, G_1_, and G_2_) and mitosis (M) phase, since aging cells may show a series of premature phenotypes including a decreased percentage of S-phase cells but increased G- and M-phase cells (Zou et al., 2019). We found that the percentage of cells in both S and G_2_ phases decreased in aging group compared to the control group, and *APOE* inhibition by siRNA increased the proportions of S and G_2_ levels, indicating that *APOE* could alleviate S arrest to some certain extent (Fig. 4J and 4K).

**Figure 5.**
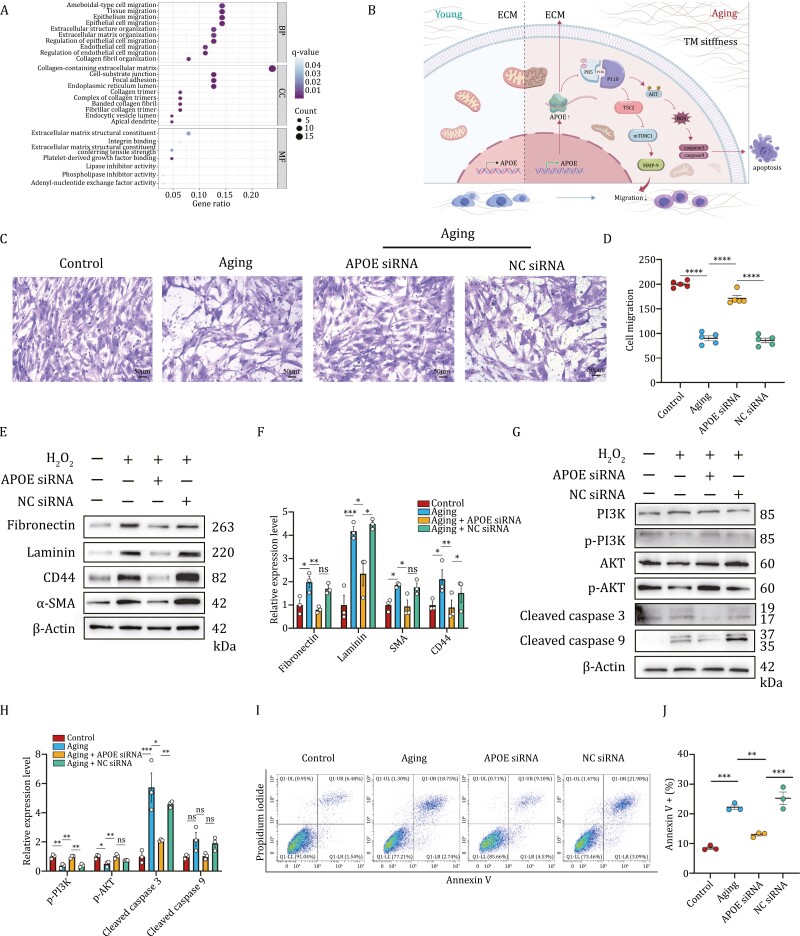
Knockdown of APOE partially rescues TMCs degeneration with aging. Functional enrichment analyses in biological process, cellular component, and molecular function of cluster 0. Schematic diagram illustrating possible mechanisms of *APOE* in trabecular meshwork degeneration with aging. TMCs were transfected with 50 nmol/L *APOE* siRNA or negative control (NC) siRNA for 24 h, followed by stimulation with H_2_O_2_ for 2 h and replacing fresh medium for more than 24 h. Transwell assay was used to investigate the migration ability of TMCs. (D) Quantification of cell migration in (C). (E) Western blot was performed to detect the protein levels of extracellular matrix (ECM) components including fibronectin, laminin, CD44, and α-SMA under different cell treatments. (F) Quantification of relative protein levels in (E). (G) Western blot was performed to detect the key molecular levels of PI3K-AKT pathway and the downstream caspase 3/9 under different cell treatments. (H) Quantification of relative protein levels in (G). (I) The apoptosis rate of TMCs in four groups was measured by flow cytometry. (J) Quantification of apoptotic proportion in all cells from (I). Data are presented as mean ± SEM values; *P < 0.05, **P < 0.01, ***P < 0.001; *n* = 3. ns: no significance.

Given that the GO analysis for cluster 0 showed that biological process (BP), cellular component (CC), and molecular function (MF) were mainly enriched in tissue migration, epithelium migration, extracellular structure organization, endothelial cell migration, collagen fibril organization, cell–substrate junction, and so on (Fig. 5A), the migration ability of TMCs and changes of ECM contents under specific stimulations were conducted respectively. Transwell assay revealed that aging cells performed decreased ability to migrate, whereas the phenomenon was reversed by *APOE* silence (Fig. 5C and 5D). The result cohered with DEG analysis and GO report of cluster 0 that *APOE* regulated a series of cellular processes and thus acting as an accelerator to induce TMC senescence. Western blot analysis was conducted to verify the expression of ECM acquired from RIPA. In contrast with the control group, upregulation expression of partial ECM constituents including fibronectin, laminin, CD44, and α-SMA were observed in aging TMCs. *APOE* siRNA treatment repressed aging-induced ECM composition transition obviously while incubation with NC siRNA didn’t rescue this conversion at all (Fig. 5E and 5F).

PI3K/AKT signaling pathway was reported to play an important role in metabolic functions such as migration, cell cycle, apoptosis, protein synthesis, and so on (Chen et al., 2022; Franke, 2008). Additionally, *APOE* has been shown to result in the inhibition of cell migration and proliferation via binding to specific cell surface receptors (Hui and Basford, 2005). To investigate whether the protective effect of *APOE* knockdown was reliant on PI3K-AKT pathway activation or suppression and thus alleviating expression of cell apoptotic markers, we performed further verification. Results of Western blot indicated that the phosphorylation level of PI3K and AKT decreased in aging cells, which was counteracted by *APOE* siRNA, releasing the deterioration of cell apoptosis due to aging, as evidenced by the reduced expression of caspase 3 and 9, the canonical apoptotic biomarker (Fig. 5G and 5H). To further clarify the role of *APOE* played in apoptosis, flow cytometry was conducted. Consistent with the results shown in Western blot, use of *APOE* silence triggered relieved apoptosis of TMCs exposed to a sublethal-dose of H_2_O_2_ (Fig. 5I and 5J). Taken together, we suppose that *APOE* could accelerate the aging process of TMCs by inducing cell apoptosis in a PI3K-AKT-dependent pathway, promoting the accumulation of ECM and restraining cell migration, thus offering inspiration for some aging-related diseases such as POAG (Fig. 5B).

### *APOE* inhibition alleviates the process of aging by improving cell apoptosis and ECM reconstruction in the mouse model

**Figure 6.**
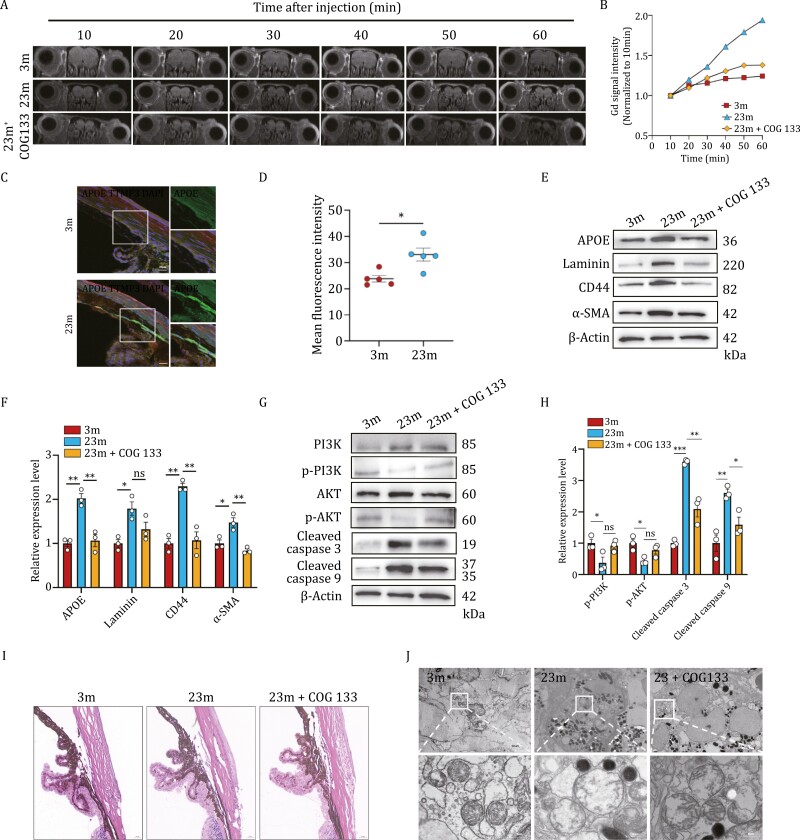
Inhibition of APOE alleviates the dysfunction of trabecular meshwork by relieving apoptosis and ECM deposition ***in vivo*.** (A) Gd-MRI was used to estimate the outflow rate of aqueous humor (AH) among 3-month, 23-month, and COG 133 treated 23-month mice. (B) Curve chart of Gd signal of different time points. (C) immunofluorescence staining was performed to detect the protein levels of APOE in mice trabecular meshwork tissues. (D) Quantification of fluorescence intensity levels in (C). (E and G) A group of 23-month-old C57BL/6 mice were intraperitoneally injected with COG 133 TFA. Mice trabecular meshwork were obtained post-treatment. Western blot was performed to detect protein levels of ECM components as well as PI3K-AKT pathway and the downstream caspase 3/9 of mice trabecular meshwork tissues. (F and H) Quantification of relative protein levels in (F and H). (I) H&E staining was used to compare the cell number, ECM of trabecular meshwork, and the angle of anterior chamber. (J) Transmission electron microscopy (TEM) was used to observe the mitochondrial changes in mice trabecular meshwork. Scale bar for H&E staining: 20 μm. Data are presented as mean ± SEM values; *P < 0.05, **P < 0.01, ***P < 0.001, ****P < 0.0001; *n* = 5 for MRI and immunofluorescence. Referring to Western blot assays, *n* = 3.

To investigate the role of *APOE* in aging animal models, we treated aging mice with a specific competitive inhibitor COG 133 TFA (COG 133). Given the crucial role of the trabecular meshwork in the drainage of aqueous humor (AH) thus maintaining a normal intraocular pressure (IOP), we conducted Gd-MRI to compare the TM function among mice at 3-month, 23-month, and 23-month after accepting COG 133 for 28 days. The results demonstrated that there was no significant difference in the Gd signal intensity of the anterior chamber during the first 20 min, but an obvious decrease of AH outflow was observed by the accumulated Gd signal another 10 min later in 23-month mice compared with young group. However, we observed a highly reduction of the contrast signal in COG 133 treated aging mice from that of aging mice (Fig. 6A and 6B), indicating the effect of *APOE* to increase AH outflow resistance *in vivo*. Next, we decided to further study the expression level of APOE in the trabecular meshwork from young and aging mice, the results suggested that the fluorescence intensity labeling APOE enhanced in 23-month mice in comparison to 3-month mice (Fig. 6C and 6D). The result was verified by Western blot and the inhibition efficacy of COG 133 was also determined (Fig. 6E and 6F). To investigate the changes in ECM components and the function of the PI3K-AKT pathway in aging and young trabecular meshwork tissues, we used Western blot to compare the expression level of corresponding molecules. Consistent with outcomes of primary TMCs under different treatments, the protein expression of laminin, CD44, α-SMA, and cleaved caspase 3/9 increased while p-PI3K and p-AKT decreased. However, after the injection of a specific inhibitor targeting on *APOE*, it promoted the protein levels of p-PI3K and p-AKT, reduced some ECM contents and cleaved caspase 3/9, which strengthened the reliability of this study (Fig. 6G and 6H).

To assess the morphological changes in the animal trabecular meshwork at both macroscopic and microscopic levels, we conducted H&E staining and TEM analysis. H&E staining revealed less TM cell nuclei which could be alleviated by COG 133 since more TMC nuclei could be seen around the Schlemm’s canal (Fig. 6I). Since the bioinformatic analysis of TMCs and *in vitro* experiments both pointed out the role of ATP synthesis and apoptosis in aging, we targeted to investigate the mitochondrial morphology changes among three groups of mice. TEM analysis confirmed more distorted mitochondrial crest, swell, and density reduction in aging mitochondria, and these abnormalities seemed to be slightly improved by *APOE* inhibition (Fig. 6J). Taken together, our results suggest that *APOE* played an important role in attenuating tissue degeneration with aging. The inhibition of APOE reversed the development of aging.

## Discussion

The goal of this study was to examine the distinct gene expression patterns in TM cells between young and old macaques and their relationship with glaucoma, as well as to explore the aging mechanism of TM in non-human primates. With the application of single-cell transcriptome sequencing technology, we identified 12 distinct cell subtypes within the outflow tissue and subsequently categorized TM cells into 13 cell clusters. The number of TM cells decreased in the aging group, while the number of macrophages and myofibroblasts increased.

Within the different cell clusters, cluster 0 displayed the most significant difference, and gene ontology analysis indicated that this subtype was associated with increased extracellular matrix composition, decreased migration ability, and apoptosis. A deeper investigation of aging datasets exposed that *APOE*, *Clu*, and *VEGFA* were the most prominent genes distinguishing between old and young macaque TM. Among these, *APOE* played a key role in the aging process, and its pathological influence was mediated through the PI3-Akt pathway. Silencing the *APOE* gene with small interfering RNA improved the aging process in both cell and animal experiments. These results provide insights into the potential pathological mechanism of glaucoma, which aligns with the increased incidence of POAG with age.

Glaucoma is an age-related disease with an incidence rate that increases with age. Structural changes in the eye, structure of elderly people, particularly in components like the trabecular meshwork and angle structure, are major factors that lead to increased intraocular pressure (Kass et al., 2002). The trabecular meshwork and Schlemm’s canal are crucial drainage channels for the aqueous humor in the anterior chamber of the eye and are the primary sites of glaucoma. The trabecular meshwork is a mesh-like structure that regulates the flow of aqueous humor and intraocular pressure, while Schlemm’s canal is the main channel for the trabecular meshwork to drain aqueous humor (Chen et al., 2018). With age, the morphology and structure of the trabecular meshwork and Schlemm’s canal may undergo alterations. The number and size of trabecular meshwork cells gradually decrease. In this study, the proportion of trabecular meshwork cells in the total outflow channel cells decreased from 45.6% to 42.5% in the old group compared to the young group. This indicates that the decrease in TM cells and the increase in smooth muscle-related cells may lead to a decrease in the elasticity of the trabecular meshwork, slowing the flow of aqueous humor, and ultimately causing a gradual decrease in the diameter and circumference of Schlemm’s canal, which further affects intraocular pressure fluctuations.

The single-cell sequencing research related to TM has established a cell atlas of the outflow pathway of the aqueous humor in the eyes of humans and four animal models. This breakthrough study aims to reveal the mechanism of glaucoma pathogenesis. Van Zyl et al. (2020) conducted a similar study, which focused on multiple species of ocular tissue by cell isolation and RNA sequencing. Their study’s findings demonstrated notable variations in the cell counts within the aqueous humor outflow pathway among humans and various species, encompassing diverse cell types. They also discovered new potential targets and biomarkers, such as the expression of genes *TGM2* and *C1QA*, which are closely related to the occurrence and development of glaucoma. Other studies have shown that endothelial cells in the TM tissue are one of the main cell types involved in aqueous humor outflow and intraocular pressure fluctuation. Their gene expression profile includes key molecules such as *AQP1*, *NOS3*, *SLC4A4*, and *SLC4A11*. The gene expression spectrum of the fibroblast subset includes a series of molecules related to extracellular matrix synthesis and regulation, such as *COL1A1*, *COL3A1*, and *SPARC* (Patel et al., 2020). All these associated molecules play a crucial role in maintaining the normal structure and physiological function of the aqueous humor outflow pathway. Our study builds upon previous research by identifying several subpopulations of cells involved in the aqueous humor outflow tissue, including TM cells, vascular pericytes, perivascular cells, myofibroblasts, endothelial cells in the adjacent vascular area, corneal-scleral cells, and Schlemm’s canal endothelial cells. Compared to previous studies, we have expanded the identification of non-human primates’ aqueous humor outflow pathway from nine cell subpopulations to 12. Additionally, we have discovered the involvement of immune cells, including macrophages, B cells, and T/NK cells.

As glaucoma originates from the TM net and the proportion of TM cells changes significantly between the elderly and young groups, our study focuses on this cell cluster. We have identified cluster 0 as the most significantly different cell cluster between the elderly and young groups, which is related to cell migration and extracellular matrix synthesis. Furthermore, we observed significant upregulation of APOE, Clu, and VEGF in the elderly group. During the clustering analysis, it was evident that APOE exhibited a higher expression level in TM cells. Therefore, we focused on its mechanism and pathway in the aging process of TM cells. *APOE* is a plasma lipoprotein primarily involved in cholesterol and lipid metabolism. Research has demonstrated that the *APOE*4 subtype is significantly associated with age-related neurodegenerative diseases such as Alzheimer’s disease, Parkinson’s disease, and Huntington’s disease (Patel et al., 2020). Since glaucoma is also a type of neurodegenerative disease, its mechanism of nerve damage has many similarities to that of degenerative diseases represented by Alzheimer’s disease, mainly manifested as neurodegenerative changes in the cerebral cortex, including neuronal loss, synaptic loss, and nerve fiber damage. Additionally, genetic studies have shown that the *APOE*4 allele is significantly positively correlated with the risk of primary angle-closure glaucoma (Vavvas et al., 2013). Some studies have indicated that *APOE* can suppress cell migration and proliferation. Hui and Basford (2005) found that in terms of cell proliferation, *APOE* binds to cells via HSPG, leading to the downregulation of the *PI3K*/*AKT*/*mTORC1* signaling pathway and consequent inhibition of cell proliferation. Regarding cell migration, *APOE* attaches to cells via LRP1, inhibiting Rac1 activation and disrupting microtubule and actin cytoskeleton in the cell’s cytoskeleton structure, as well as decreasing the expression of cell surface adhesion molecules, all contributing to the suppression of cell migration.

The *PI3K*/*AKT* signaling pathway is a widely involved signaling pathway in various biological processes such as cell proliferation, growth, metabolism, and differentiation. It plays a crucial regulatory role in metabolic regulation, cell cycle, cell adhesion, and cell migration (Engelman, 2009). Activation of *AKT* by the *PI3K-AKT* pathway promotes cell growth and division by phosphorylation and regulation of various effectors. Additionally, it has an inhibitory effect on cell apoptosis and death processes. *AKT* promotes cell migration and invasion by phosphorylating and activating various proteins such as GSK-3β, paxillin, and Myosin. Simultaneously, this pathway can also modulate the expression of matrix metalloproteinases (MMPs), consequently influencing cell adhesion and invasion into the matrix (Fruman et al., 2017). In this study, we observed higher expression of APOE in the aging group compared to the young group. Therefore, upon inducing the aging phenotype in primary trabecular meshwork cells, we detected inhibition of the *PI3K-AKT* pathway and activation of Caspase, resulting in cell number. At the same time, cell cycle experiments revealed a diminished capacity for proliferation and division in the aging group cells were weakened, which is consistent with our previous conclusion that functional alterations occur during cell aging (Childs et al., 2014; López-Otín et al., 2023). One of the “hallmark features” of aging is the decline in cell proliferation and repair abilities, which can result in cell apoptosis due to their weakened proliferation ability. When we silenced the *APOE* gene by small interfering RNA, we observed an upregulation of the expression levels of *PI3K* and *AKT* molecules, and an increase in the migration and proliferation abilities of trabecular meshwork cells, suggesting that *APOE* is involved in the regulation of the physiological functions of trabecular meshwork cells and improves their aging phenotype.

In addition, the extracellular matrix (ECM) is a vital component of aqueous humor outflow tissue and plays a crucial role in the regulation of intraocular pressure. The ECM of the TM is a complex network structure composed of various molecules such as collagen, elastin, and fibronectin (Theocharis et al., 2016). Abnormal deposition of ECM in the TM may lead to a decrease in its migratory ability. Enhanced deposition and sclerosis of the extracellular matrix (ECM) within the trabecular meshwork (TM) have been proposed as potential factors leading to elevated intraocular pressure in glaucoma patients (Rhee et al., 2009). The results of this study indicate that the levels of fibronectin, laminin, *CD44*, and *α-SMA* were upregulated in the ECM of aged TM cells. *In vivo* and *in vitro* knockout of the APOE gene led to a substantial reduction in the expression levels of these components, suggesting that *APOE* participates in the regulation of ECM components through its interaction with ECM proteins. Studies have demonstrated that *APOE* can interact with various ECM proteins, cell surface–specific proteins, and glycoproteins such as heparan sulfate proteoglycans (HSPGs), collaborating to regulate the metabolism and degradation of ECM (Mahley and Huang, 2012). Recent research findings indicate that *APOE* knockout mice exhibit reduced optic nerve axonal damage and weaker retinal glial cell responses. Subsequent molecular mechanism studies have shown that APOE4 inhibits the expression of microRNA-124 (miR-124), which reduces the neurotransmitter release of glial cells, promotes apoptosis and migration, and promotes the repair and regeneration of optic nerve damage (Margeta et al., 2022).

While the findings of this study are instructive, there are still some limitations and challenges: First, the experimental design of this non-human primate eye disease study only included middle-aged and old macaques, lacking a younger group. In the future, the sample size should be expanded to enrich the research results and strengthen our conclusions. Meanwhile, *GLU* and *VEGFA* genes also exhibit strong age-relatedness. more comprehensive research is needed to improve the study of other TM aging-related genes and signaling pathways in the further study. In addition, it is essential to note that the findings of this study are derived from experiments involving the silencing of the APOE gene, primarily conducted on non-human primates. In the future, it is necessary to expand to other animal models for further research to determine their therapeutic effects and side effects (effectiveness and safety).

Our study offers initial insights into the changes occurring in the body and genetic levels during the aging of the macaque TM. This is of significant importance for comprehending the aging mechanism of the macaque TM and the pathogenesis of related diseases and providing vital clues for comprehending the pathological mechanism of glaucoma. By employing single-cell transcriptome sequencing and employing siRNA to silence the APOE gene, we demonstrated its potential as a therapeutic target. These findings underscore the potential for the development of novel treatment strategies for glaucoma and other TM-related diseases.

## Methods

### Ethics statement

This study was authorized by the Beijing Tongren Hospital of Capital Medical University. The experimental procedures of primates and mice were all carried out in accordance with the guidelines of the ARVO Statement of Animals in Ophthalmic and Vision Research with approval from the Ethics Committee for Animal Studies of Beijing Tongren Hospital of Capital Medical University.

### Cell lines and cell cultures

Primary human trabecular meshwork cells (TMCs), isolated from the juxtacanalicular and corneoscleral regions of human eye, were obtained from the Sciencell corporation (ScienCell, Carlsbad, CA, USA). TMCs were cultured in TMCM medium (ScienCell), containing 2% fetal bovine serum (ScienCell), 1% penicillin/streptomycin (ScienCell) as well as 1% TM cell growth supplement (ScienCell). The cells were maintained in a 37°C, 5% CO_2_ incubator.

U937 cell line was isolated from the pleural fluid of a patient with histiocytic lymphoma, which was purchased from Procell Life Science & Technology (Wuhan, China). The cells were cultured in RPMI 1640 supplemented with 10% fetal bovine serum (164210-50, Procell) and 1% penicillin/streptomycin (PB180120, Procell) at 37°C with 5% CO_2_.

### Mice

Three-month and 23-month male wildtype C57BL/6J mice purchased from Wukong Biotechnology (Jiangsu, China) were used in the study. All animals were housed and fed in a specific-pathogen-free (SPF) condition at an animal facility in Capital Medical University with a regular 12-h light/dark cycle, 40%–60% humidity, and a proper constant temperature of 19–22°C. To inhibit the expression of APOE in aging mice, we used the specific competitive inhibitor COG 133 TFA (HY-P1050A, MedChemExpress, New Jersey, USA) to perform an intraperitoneal injection at a dose of 1 mg/kg once every two days for 28 days, other groups were administrated with the same quantity of sterilized phosphate buffered saline (PBS).

### Induction of TMCs senescence

Before induction of TMCs senescence, CCK-8 assay was conducted to analyze the cell viability under different amounts and durations of hydrogen peroxide (H_2_O_2_). The result is shown in Fig. S5A. To induce TMCs aging under oxidative stress, the sublethal concentration of H_2_O_2_ (50–100 μmol/L) was added into the medium for 2 h. Then the cells were washed with PBS to terminate the effect of H_2_O_2_ and cells were cultured in normal TMCM medium for more than 24 h.

### Induction of U937 cell line senescence

4β-phorbol-12-myristate-13-acetate (PMA) (79346, Sigma-Aldrich, St. Louis, MO, USA) at a concentration of 100 ng/mL was applied to differentiate U937 cells into macrophages. After incubation for 24 h, the cells were stimulated with 1 μg/mL Lipopolysaccharide (LPS) for senescence induction.

### Small interfering RNA

Human APOE siRNA and negative control (NC) siRNA were purchased from Ribobio (Guangzhou, China). Prior to H_2_O_2_ stimulation, TMCs at 30%–50% confluency were transfected with 50 nmol/L APOE siRNA or NC siRNA using riboFECT™ CP Transfection kit (C10511-05, Ribobio) for 24 h according to the manufacturer’s instruction. The sequence of the siRNA was CTGGGTCGCTTTTGGGATT.

### SA-β-gal staining

SA-β-gal staining was performed according to the manufacturer’s instructions (C0602, Beyotime, Shanghai, China). The medium was discarded and TMCs were washed with PBS once, fixed for 15 min, and incubated with certain quantity of blended staining solution at 37°C without CO_2_ overnight. The cells were observed under the optical microscope (Leica DMi8, Germany).

### Quantitative real-time PCR (qRT-PCR)

The total RNA of TMCs was extracted by TRIzol reagent (15596018, Invitrogen). The purity and concentration of RNA were detected by Nanodrop 2000 (Thermo Scientific, USA). RNA (1 μg) was applied for synthesizing first-strand cDNA using the GoScript™ Reverse Transcription System (A5000, Promega, Madison, Wisconsin, USA). PowerUp™ SYBR™ Green Master Mix (A25742, Applied Biosystems, USA) was used to perform real-time PCR with the 7500 Real Time PCR System (Applied Biosystems, Waltham, MA, USA). The primer sequences of *APOE*, *CLU*, *VEGFA,* and *β-actin* are shown in Table 1. The relative expression level of target genes was normalized to that of *β-actin* using the 2^−ΔΔCt^ method. There were three replications for each group.

**Table 1.** Primer sequences for mRNA expression analysis.

Gene names	Forward primer, 5ʹ-3ʹ	Reverse primer, 3ʹ-5ʹ
*APOE*	TGGAGCAAGCGGTGGAGACAG	AAAAGCGACCCAGTGCCAGTTC
*Clu*	GCCATGTTCCAGCCCTTCCTTG	GGTCATCGTCGCCTTCTCGTATG
*VEGFA*	CTTCGCTTACTCTCACCTGCTTCTG	GCTGTCATGGGCTGCTTCTTCC
*β-Actin*	CAGATGTGGATCAGCAAGCAGGAG	CGCAACTAAGTCATAGTCCGCCTAG

### Western blot

C56BL/6 mouse trabecular meshwork tissues were gently dissected from corneoscleral limbus. The proteins of tissues and TMCs were harvested using radio-immunoprecipitation assay (RIPA) buffer (Thermo, Waltham, USA) supplemented with protease inhibitors. About 30 μg protein was separated using 8%–12% sodium dodecyl sulfate-polyacrylamide gel electrophoresis (SDS-PAGE), then transferred onto the polyvinylidene fluoride membranes (Merck Millipore, Burlington, MA, USA) and blocked in 5% nonfat milk for 1 h. Subsequently, the membranes were incubated with primary antibodies at 4°C overnight and corresponding secondary antibodies for 1 h at room temperature. The chemiluminescence reagent kit (CWBIO, Beijing, China) was subjected to the membrane for visualization of blots. The protein expression levels were measured by Image J 1.52a software. The antibodies against APOE (13366) and β-galactosidase (i.e., SA-β-gal, 27198) were obtained from Cell Signaling Technology (Danvers, MA, USA). Antibodies against CLU (12289-1-AP), VEGFA (19003-1-AP), P21 (10355-1-AP), laminin (23498-1-AP), CD44 (15675-1-AP), PI3K (60225-1-Ig), AKT (10176-2-AP), p-AKT (Ser473, 66444-1-Ig), caspase 3 (19677-1-AP) and caspase 9 (10380-1-AP) were purchased from Proteintech (Wuhan, China). Antibody for p-PI3K (Tyr607, CY6427) was purchased from Abways (Shanghai, China). Antibodies for fibronectin (ab32419) and α-SMA (A5228) were separately from Abcam (Cambridge, UK) and Sigma-Aldrich (St. Louis, MO, USA). The secondary antibodies, anti-Rabbit IgG (7074) and anti-Mouse IgG (7076) were obtained from Cell Signaling Technology.

### Immunofluorescence double staining

The localization and expression of different proteins were estimated using immunofluorescence staining. TMCs were seeded onto glass slides with a diameter of 14 mm at a number of 2 × 10^4^ and treated as described above. The trabecular meshwork tissues were dissected and fixed with 4% paraformaldehyde (PFA) for 2 h followed by 30% sucrose at 4°C overnight. Fixed tissues were embedded in Tissue-Tek optimal cutting temperature (O.C.T.) compound (SAKURA, Tokyo, Japan) and sectioned into 15 μm slices using Leica freezing microtome (Leica CM3050 S, Nussloch, Germany).

TMCs were fixed with 4% paraformaldehyde (PFA) for 25 min and subsequently 0.1% Triton X-100 for 20 min at room temperature. TMCs and sectioned trabecular meshwork tissues were blocked with 3% bovine serum albumin (BSA) (GC305006, Servicebio, Wuhan, China) for 1 h at room temperature. Then the sections and cultured cells were incubated simultaneously with TIMP3 primary antibody (1:50, Santa Cruz biotechnology, Dallas, TX, USA) and APOE, CLU, VEGFA, p21, β-galactosidase primary antibody (1:100) at 4°C overnight, they were then washed with PBS and further incubated with Alexa Fluor 488- or 594-conjugated anti- Rabbit/Mouse secondary antibodies (1:500, Invitrogen, Waltham, MA, USA) for 1 h at room temperature. Nuclei were stained with DAPI (Sigma-Aldrich, USA).

Photographs were captured with a confocal microscope (Zeiss LSM 900). Fluorescence intensity was analyzed using Image J 1.52a software.

### Migration assay

Migration assay was launched using Transwell systems in a 24-well plate (Corning Costar, Cambridge, MA, USA). TMCs of each group were digested and seeded in the upper chamber with the amount of 5 × 10^4^ in 100 μL DMEM/F12 medium (D8437, Sigma-Aldrich, St. Louis, MO, USA) without serum, and the lower chamber was added with 500 μL TMCM medium. The cells were washed gently with PBS, fixed with 4% PFA for 20 min, and stained with 0.1% crystal violet (DM0119, Leagene Biotechnology, Beijing, China) for 20 min. The Transwell chambers were washed three times and the cells on the inner membrane were wiped off with wet cotton swabs. The photos were captured with Leica DMi8 (Germany) and five randomly microscopic fields were selected for analysis.

### Cell cycle

The cell cycle measurement was performed using Cell Cycle Detection Kit (KGA-511, Keygen Biotech, Nanjing, China). The cells were collected, washed with cold PBS, and fixed with 70% cold ethanol at 4°C overnight. The cells were washed the next day. Then the cells were treated with 50 μL RNase and incubated for 30 min at room temperature, finally the cells were stained with 450 μL PI. Cell cycle rates were recorded and analyzed with Kaluza Analysis 2.1 software.

### Cell apoptosis

The Annexin V-FITC/PI apoptosis detection kit (A211-01, Vazyme, Nanjing, China) was applied for quantifying the apoptosis of TMCs according to the manufacturer’s instructions. Briefly, cells were digested and washed with cold PBS for three times, then the cells were suspended with 100 μL 1× binding buffer. 5 μL Annexin V-FITC and 5 μL PI staining solution were added and incubated for 10 min at room temperature away from light, then 400 μL 1× binding buffer was added and the mixture was subjected to flow cytometry.

### ATP assay

The ATP production after cell treatment was assessed using ATP Assay Kit (S0026, Beyotime, Shanghai, China). TMCs were lysed with the lysis buffer, then the supernatant was acquired after centrifuge at 12,000 ×*g* at 4°C for 5 min, and 40 μL samples were added to 100 μL ATP detection working solution. Then the luciferase intensity was measured by luminoscence spectrometry (TECAN Spark®, Switzerland). The concentration of ATP was calculated according to the standard curve plotted.

### Anterior segment optical coherence tomography (AS-OCT) and Slit lamp examination

AS-OCT was performed with Ultramicro Ophthalmol Imaging System (Heidelberg Engineering, Heidelberg, Germany). Mice were anesthetized with 240 mg/kg Tribromoethanol (T48402, Sigma-Aldrich, St. Louis, MO, USA) and placed properly. Then promethazine hydrochloride and the carbomer eye drops were applied to the cornea. The images of mice anterior chambers for both eyes were captured after optical source of the machine was focused on pupils. The angle mode was applied for each photography. Central anterior chamber depth was tested by Image J 1.52a software with low-quality images excluded. Slit lamp (BX-900, Haag-Streit AG, Koeniz, Switzerland) examination was conducted subsequently to observe the anterior segment of mice with both the diffused and slit light. Photographs were captured accordingly.

### Gadolinium magnetic resonance imaging

Gd-MRI was applied to observe the aqueous humor (AH) dynamics as mentioned previously (Nair et al., 2021; Yan et al., 2022). Briefly, mice were anesthetized with 2% isoflurane and kept warm with their respiration rate monitored carefully by a small pneumatic pillow. They were placed in a proper position to acquire the baseline data followed by intraperitoneally injection of Gadolinium-DTPA with the dose of 0.3 mmol/kg. RARE-T1-weighted MRI signals were recorded every 10 min for 1 h. Coronal, sagittal, and transverse positions were all scanned for localization and interesting shoots were chosen for the final semi-quantitative analysis. Scanning parameters were as follows: field of view = 14.4 × 14.4 mm^2^, repetition time/echo time = 600/9 ms, and in-plane resolution = 256 × 256 μm^2^. The average gray intensity of contrast medium in the anterior chamber was measured using Image J 1.52a software and the time-to-intensity curve was depicted with GraphPad Prism 9.0.0.

### Hematoxylin and eosin staining

To evaluate the histological characteristics of trabecular meshwork and anterior chamber angle of mice from different groups, animals were cervical dislocated and the eyes were enucleated and fixed in 4% paraformaldehyde for 2 h at room temperature. Then the samples were washed with PBS, dehydrated in gradient ethanol, and embedded in paraffin. Tissues were sliced with a thickness of 5 μm and stained with hematoxylin and eosin.

### Transmission electron microscope (TEM)

TM tissues were separated and dissected into 2 × 2 mm slices. Then the samples were fixed with 2.5% glutaraldehyde and 3% osmium tetroxide subsequently at room temperature. After dehydration in gradient ethanol, the tissues were embedded in epoxy resin and cut in ultrathin (~60–80 nm) sections. Finally, the images were acquired at 3.0 K and 20.0 K magnifications using the Hitachi TEM system (HT7800, Tokyo, Japan).

### Statistical analysis

All data were analyzed using GraphPad Prism 9.0.0 (GraphPad Software, La Jolla, CA, USA) and images were processed with Adobe Photoshop CS6 (Adobe, San Jose, CA, USA). The Student’s *t*-test was used to analyze the differences between the two groups. One-way ANOVA was used to compare differences among multiple groups. *P* < 0.05 was considered to be statistically significant. All experiments were replicated for at least three times and the data were expressed as mean ± standard error of the mean (SEM).

## Supplementary information

The online version contains supplementary material available at https://doi.org/10.1093/procel/pwad067.

pwad067_suppl_Supplementary_Figures_S1-S5

## Data Availability

All data generated or analyzed during this study are included in this published article (and its supplementary information files), further inquiries can be directed to the corresponding authors.

## References

[CIT0001] Adams CM , Stacy R, Rangaswamy N et al. Glaucoma—next generation therapeutics: impossible to possible. Pharm Res 2018;36:25. 30547244 10.1007/s11095-018-2557-4

[CIT0002] Angello JC , Pendergrass WR, Norwood TH et al. Proliferative potential of human fibroblasts: an inverse dependence on cell size. J Cell Physiol 2005;132:125–130. 10.1002/jcp.1041320117 3597549

[CIT0003] Beykin G , Norcia AM, Srinivasan VJ et al. Discovery and clinical translation of novel glaucoma biomarkers. Prog Retin Eye Res 2021;80:100875. 32659431 10.1016/j.preteyeres.2020.100875 PMC7796965

[CIT0004] Burton MJ , Ramke J, Marques AP et al. The Lancet Global Health Commission on Global Eye Health: vision beyond 2020. Lancet Global Health 2021;9:e489–e551. 33607016 10.1016/S2214-109X(20)30488-5 PMC7966694

[CIT0005] Chen Z , Sun J, Li M et al. Effect of age on the morphologies of the human Schlemm’s canal and trabecular meshwork measured with swept‑source optical coherence tomography. Eye (London, England) 2018;32:1621–1628. 29921951 10.1038/s41433-018-0148-6 PMC6189106

[CIT0006] Chen K , Li Y, Zhang X et al. The role of the PI3K/AKT signalling pathway in the corneal epithelium: recent updates. Cell Death Dis 2022;13:513. 35641491 10.1038/s41419-022-04963-x PMC9156734

[CIT0007] Childs BG , Baker DJ, Kirkland JL et al. Senescence and apoptosis: dueling or complementary cell fates? EMBO Rep 2014;15:1139–1153. 25312810 10.15252/embr.201439245 PMC4253488

[CIT0008] Cristofalo VJ , Sharf BB. Cellular senescence and DNA synthesis. Exp Cell Res 1973;76:419–427. 4568162 10.1016/0014-4827(73)90394-7

[CIT0009] Croft MA , Lütjen-Drecoll E, Kaufman PL. Age-related posterior ciliary muscle restriction—a link between trabecular meshwork and optic nerve head pathophysiology. Exp Eye Res 2017;158:187–189. 27453343 10.1016/j.exer.2016.07.007 PMC5253323

[CIT0010] de Jesus BB , Blasco MA. Assessing cell and organ senescence biomarkers. Circ Res 2012;111:97–109. 22723221 10.1161/CIRCRESAHA.111.247866 PMC4824275

[CIT0011] Dimri GP , Lee X, Basile G et al. A biomarker that identifies senescent human cells in culture and in aging skin in vivo. Proc Natl Acad Sci USA 1995;92:9363–9367. 7568133 10.1073/pnas.92.20.9363 PMC40985

[CIT0012] Engelman JA. Targeting PI3K signalling in cancer: opportunities, challenges and limitations. Nat Rev Cancer 2009;9:550–562. 19629070 10.1038/nrc2664

[CIT0013] Franke TF. PI3K/Akt: getting it right matters. Oncogene 2008;27:6473–6488. 18955974 10.1038/onc.2008.313

[CIT0014] Fruman DA , Chiu H, Hopkins BD et al. The PI3K Pathway in human disease. Cell 2017;170:605–635. 28802037 10.1016/j.cell.2017.07.029 PMC5726441

[CIT0015] Gold ME , Kansara S, Nagi KS et al. Age-related changes in trabecular meshwork imaging. Biomed Res Int 2013;2013:1–6. 10.1155/2013/295204 PMC3791583 24163814

[CIT0016] Hui DY , Basford JE. Distinct signaling mechanisms for apoE inhibition of cell migration and proliferation. Neurobiol Aging 2005;26:317–323. 15639309 10.1016/j.neurobiolaging.2004.02.030

[CIT0017] Johnson M. What controls aqueous humour outflow resistance?. Exp Eye Res 2006;82:545–557. 16386733 10.1016/j.exer.2005.10.011 PMC2892751

[CIT0018] Jonas JB , Aung T, Bourne RR et al. Glaucoma. Lancet (London, England) 2017;390:2183–2193. 28577860 10.1016/S0140-6736(17)31469-1

[CIT0019] Kass MA , Heuer DK, Higginbotham EJ et al. The ocular hypertension treatment study: a randomized trial determines that topical ocular hypotensive medication delays or prevents the onset of primary open-angle glaucoma. Arch Ophthalmol 2002;120:701–713. 12049574 10.1001/archopht.120.6.701

[CIT0020] Kumazaki T , Robetorye RS, Robetorye SC et al. Fibronectin expression increases during *in vitro* cellular senescence: correlation with increased cell area. Exp Cell Res 1991;195:13–19. 2055262 10.1016/0014-4827(91)90494-f

[CIT0021] Liu B , Mcnally S, Kilpatrick JI et al. Aging and ocular tissue stiffness in glaucoma. Surv Ophthalmol 2018;63:56–74. 28666629 10.1016/j.survophthal.2017.06.007

[CIT0022] López-Otín C , Blasco MA, Partridge L et al. Hallmarks of aging: an expanding universe. Cell 2023;186:243–278. 36599349 10.1016/j.cell.2022.11.001

[CIT0023] Macosko EZ , Basu A, Satija R et al. Highly parallel genome-wide expression profiling of individual cells using nanoliter droplets. Cell 2015;161:1202–1214. 26000488 10.1016/j.cell.2015.05.002 PMC4481139

[CIT0024] Mahley RW , Huang Y. Apolipoprotein E sets the stage: response to injury triggers neuropathology. Neuron 2012;76:871–885. 23217737 10.1016/j.neuron.2012.11.020 PMC4891195

[CIT0025] Margeta MA , Yin Z, Madore C et al. Apolipoprotein E4 impairs the response of neurodegenerative retinal microglia and prevents neuronal loss in glaucoma. Immunity 2022;55:1627–1644.e7. 35977543 10.1016/j.immuni.2022.07.014 PMC9488669

[CIT0026] Nair KS , Srivastava C, Brown RV et al. GLIS1 regulates trabecular meshwork function and intraocular pressure and is associated with glaucoma in humans. Nat Commun 2021;12. 10.1038/s41467-021-25181-7 PMC8361148 34385434

[CIT0027] Pascolini D , Mariotti SP. Global estimates of visual impairment: 2010. Br J Ophthalmol 2012;96:614–618. 22133988 10.1136/bjophthalmol-2011-300539

[CIT0028] Patel G , Fury W, Yang H et al. Molecular taxonomy of human ocular outflow tissues defined by single-cell transcriptomics. Proc Natl Acad Sci USA 2020;117:12856–12867. 32439707 10.1073/pnas.2001896117 PMC7293718

[CIT0029] Picaud S , Dalkara D, Marazova K et al. The primate model for understanding and restoring vision. Proc Natl Acad Sci USA 2019;116:26280–26287. 31871177 10.1073/pnas.1902292116 PMC6936588

[CIT0030] Rhee DJ , Haddadin RI, Kang MH et al. Matricellular proteins in the trabecular meshwork. Exp Eye Res 2009;88:694–703. 19101543 10.1016/j.exer.2008.11.032

[CIT0031] Sherwood SW , Rush D, Ellsworth JL et al. Defining cellular senescence in IMR-90 cells: a flow cytometric analysis. Proc Natl Acad Sci USA 1988;85:9086–9090. 3194411 10.1073/pnas.85.23.9086 PMC282668

[CIT0032] Stamer WD , Acott TS. Current understanding of conventional outflow dysfunction in glaucoma. Curr Opin Ophthalmol 2012;23:135–143. 22262082 10.1097/ICU.0b013e32834ff23e PMC3770936

[CIT0033] Stamer WD , Clark AF. The many faces of the trabecular meshwork cell. Exp Eye Res 2017;158:112–123. 27443500 10.1016/j.exer.2016.07.009 PMC5247412

[CIT0034] Tham Y-C , Li X, Wong TY et al. Global prevalence of glaucoma and projections of glaucoma burden through 2040. Ophthalmology 2014;121:2081–2090. 24974815 10.1016/j.ophtha.2014.05.013

[CIT0035] Theocharis AD , Skandalis SS, Gialeli C et al. Extracellular matrix structure. Adv Drug Deliv Rev 2016;97:4–27. 26562801 10.1016/j.addr.2015.11.001

[CIT0036] van Zyl T , Yan W, Mcadams A et al. Cell atlas of aqueous humor outflow pathways in eyes of humans and four model species provides insight into glaucoma pathogenesis. Proc Natl Acad Sci USA 2020;117:10339–10349. 32341164 10.1073/pnas.2001250117 PMC7229661

[CIT0037] Vavvas D , Song Q, Chen P et al. Role of the APOE ε2/ε3/ε4 polymorphism in the development of primary open-angle glaucoma: evidence from a comprehensive meta-analysis. PLoS One 2013;8:e82347. 24312416 10.1371/journal.pone.0082347 PMC3842323

[CIT0038] Weinreb RN , Leung CKS, Crowston JG et al. Primary open-angle glaucoma. Nat Rev Dis Primers 2016;2. 10.1038/nrdp.2016.67 27654570

[CIT0039] Yan X , Wu S, Liu Q et al. Myocilin gene mutation induced autophagy activation causes dysfunction of trabecular meshwork cells. Front Cell Dev Biol 2022;10. 10.3389/fcell.2022.900777 PMC9124892 35615698

[CIT0040] Zou W , Fu L, Hu Y et al. Up-regulation of FOXD1 by YAP alleviates senescence and osteoarthritis. PLoS Biol 2019;17. 10.1371/journal.pbio.3000201 PMC6459557 30933975

