# Supplementary material for: Single-cell transcriptomic Atlas of aging macaque ocular outflow tissues
Source: Protein Cell. 2024 Feb 15;15(8):594–611. doi: 10.1093/procel/pwad067 (PMC11259549; doi:10.1093/procel/pwad067)
Supplement: pwad067_suppl_Supplementary_Figures_S1-S5 [file pwad067_suppl_supplementary_figures_s1-s5.pdf]

Supplementary material

Supplementary Figure 1

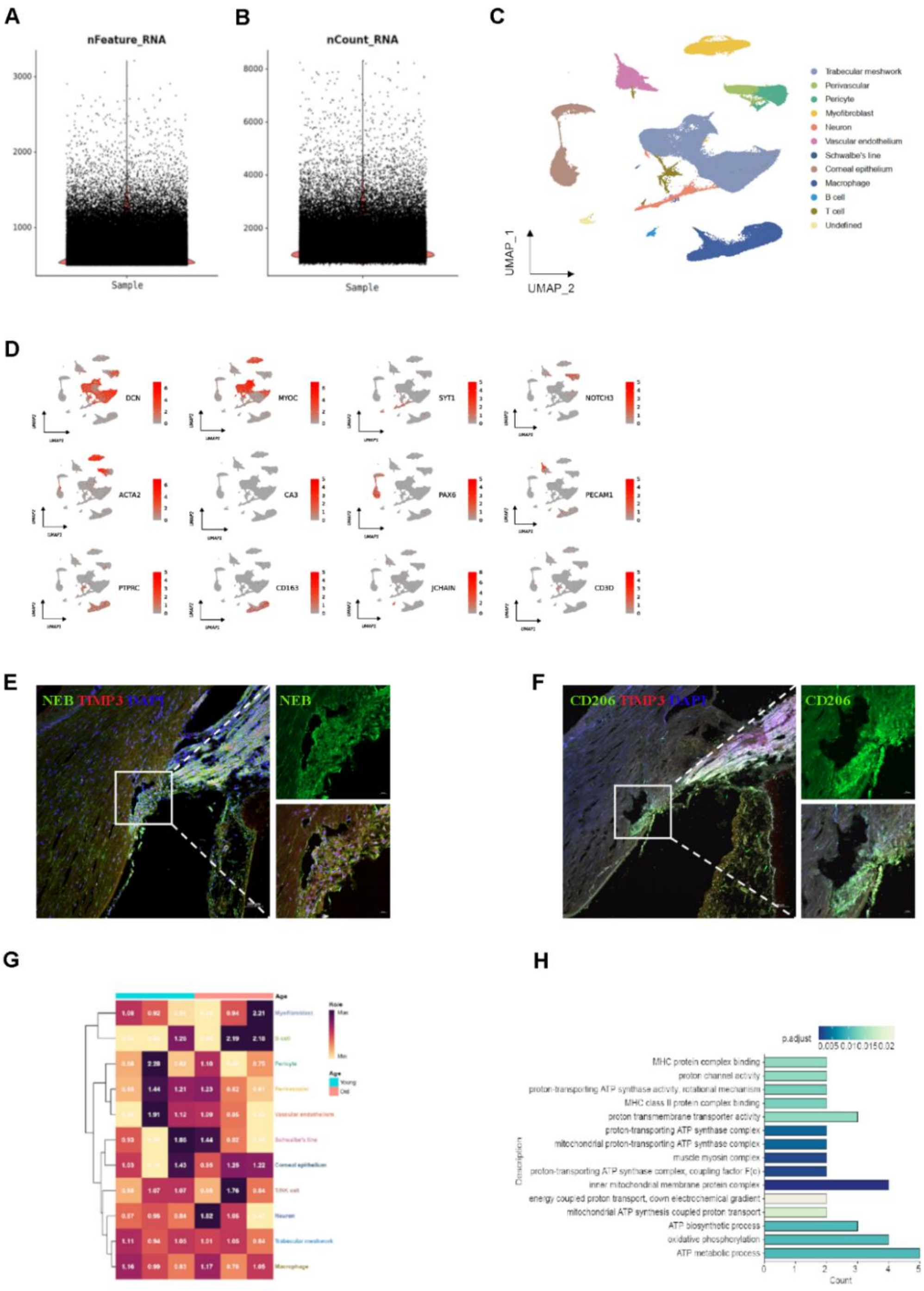

(A) DoubletFinder and FindIntegrationAnchors was used to remove batch effects and perform quality control. (B) Cell types of trabecular meshwork shown in UMAP plot. (C-D) UMAP plot of cell marker genes of trabecular meshwork. (E) Immunifluosence stainging was performed to detect the protein levels of NEB in monkey trabecular meshworktissues. (F) Immunifluosence stainging was performed to detect the protein levels of CD206 in monkey trabecular meshworktissues. (G) Heatmap illustrating the difference of these cell types between young and aging macaque trabecular meshwork that some are mainly enriched in the young group, the rest of the cells were mainly concentrated in the senescent group. (H) Genetic Ontology (GO) enrichment analysis results for all cell types of trabecular meshwork tissues in aging samples, showing the involvement of MHC protein and ATP synthase.

Supplementary Figure 2. GO analysis of the specific DEGs in each cluster in trabecular meshwork cells.

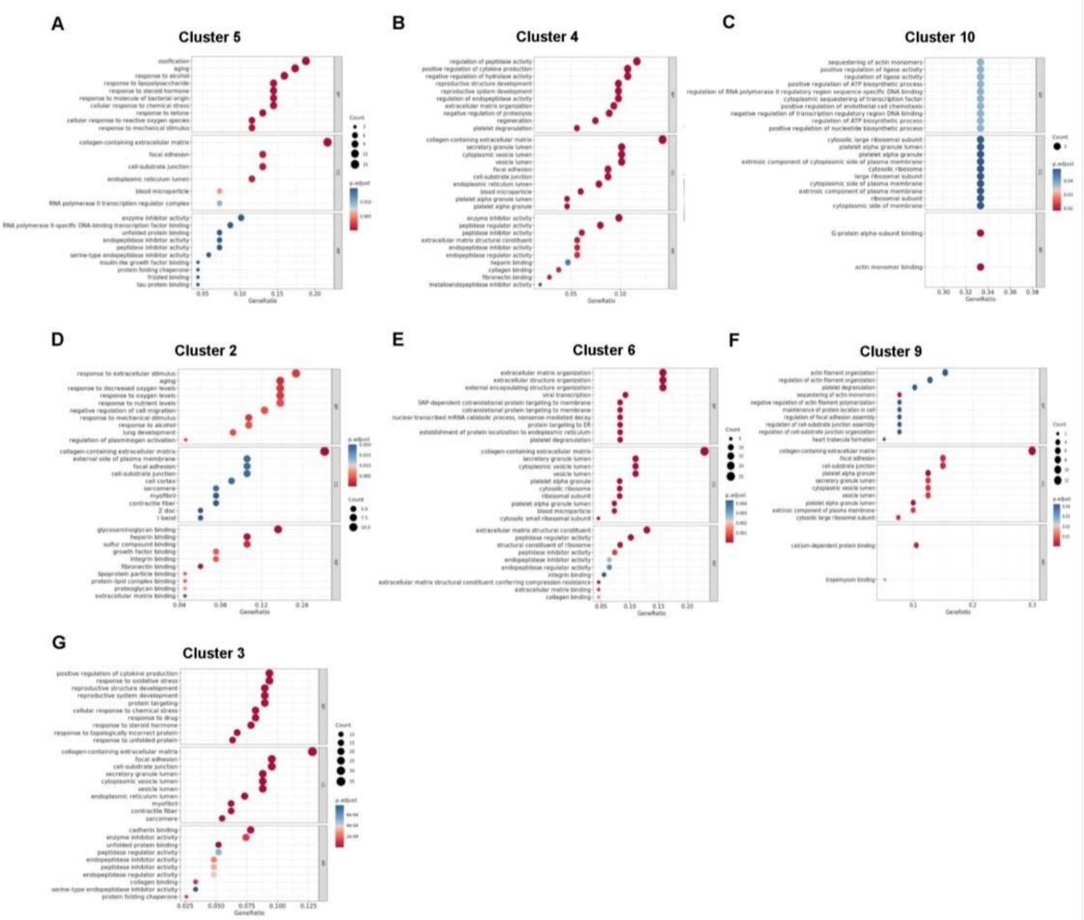

Supplementary Figure 3. KEGG analysis of the specific DEGs in each cluster in trabecular meshwork cells.

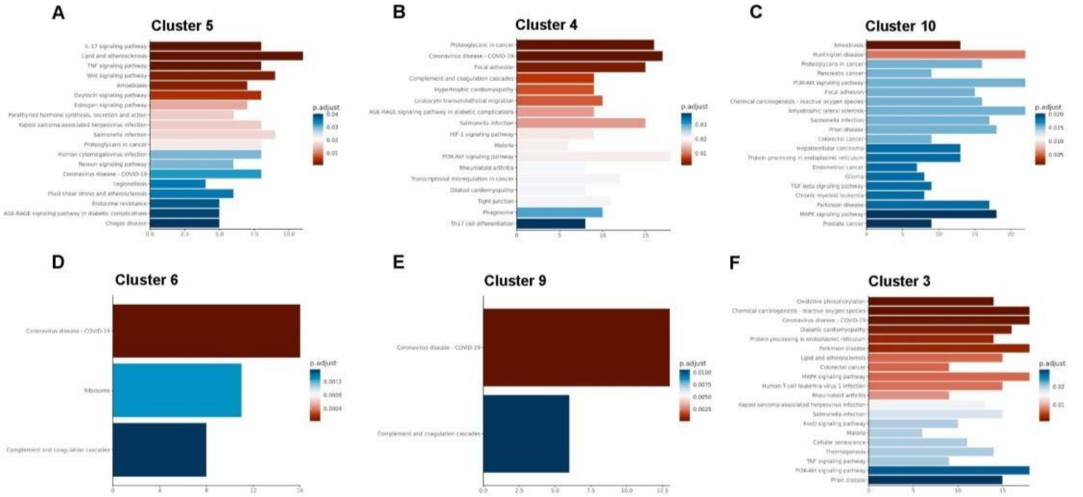

Supplementary Figure 4

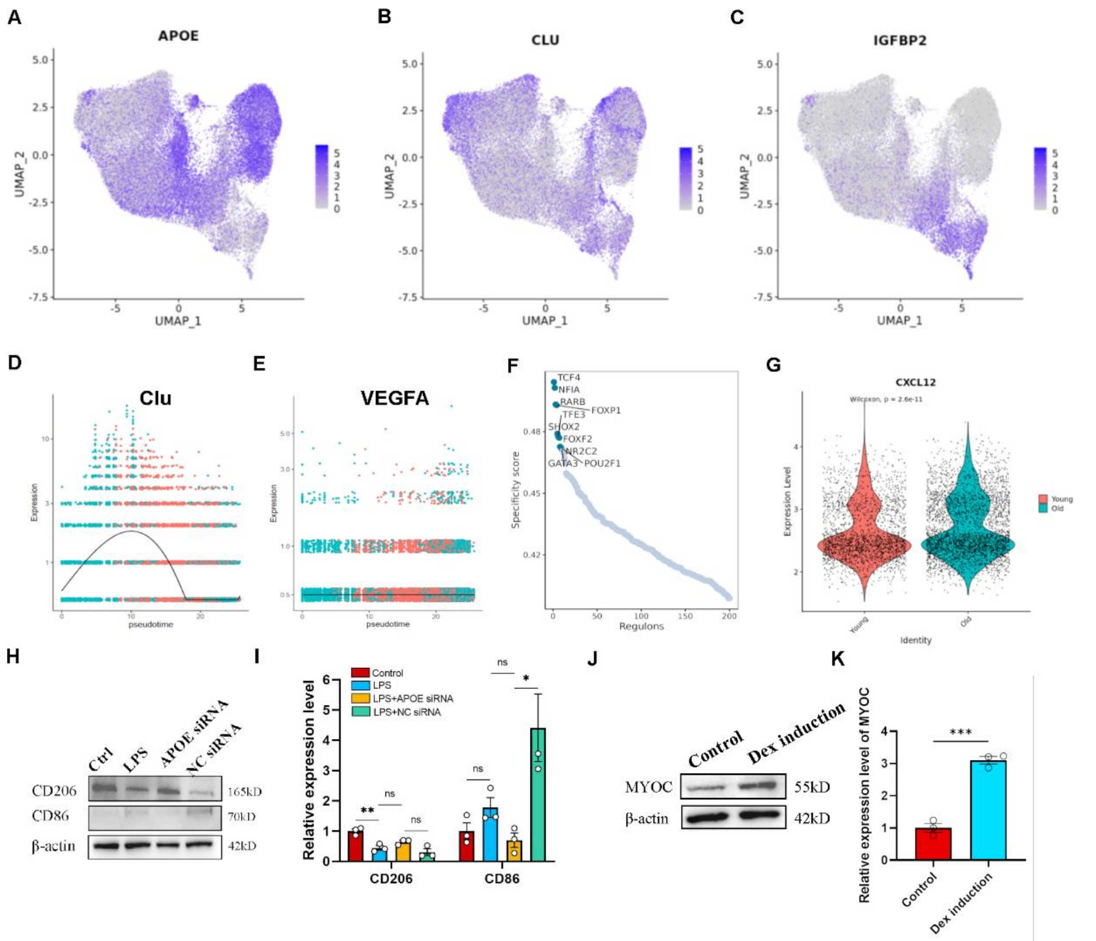

(A-C) UMAP plot showing the distribution of aging-related genes including *APOE*, *CLU* and *IGFBP2* in trabecular meshwork. (D and E) Pseudotime of cluster 0 with the effect of *CLU* and *VEGFA* generated by Monocle3package of R software. Each dot represents a single cell. (F) Specific score of regulons of *APOE* with aging. (G) Violin plot showing the expression level of CXCL12 in young and aging samples. (H and I) The expression level of M1 and M2 marker genes were validated by western blot in aging and APOE knockdown cells. (J and K) The expression level of MYOC before and after dexamethasone induction illustrated by western blot in the primary TMCs. Data are presented as mean ± SEM values; *$p < 0.05$, **$p < 0.01$, ***$p < 0.001$; n = 3

Supplementary Figure 5

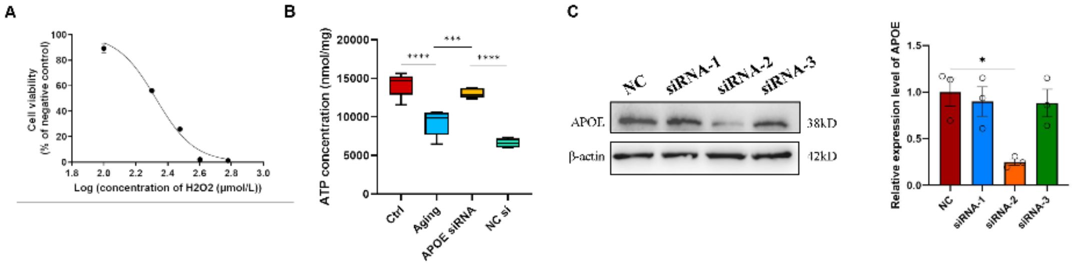

(A) The viability of TMCs with the stimulation of H2O2 was tested by CCK-8 assay. (B) TMCs were transfected with 50 nM APOE siRNA or negative control (NC) siRNA for 24h, followed by stimulation with H2O2 for 2h and replacing fresh medium for more than 24h. ATP assay was performed to detect the ATP concentration under different cell treatments. (C) Western blot was performed to examine the knock-down efficiency of three siRNA sequences. Data are presented as mean ± SEM values; *p < 0.05, **p < 0.01, ***p < 0.001, ****p < 0.0001; n = 3.
